# Supplementary material for: Hierarchical design of pseudosymmetric protein nanoparticles
Source: bioRxiv. 2023 Jun 17:2023.06.16.545393. Preprint. [Version 1] doi: 10.1101/2023.06.16.545393 (PMC10312784; doi:10.1101/2023.06.16.545393)
Supplement: Supplement 1 [file media-1.pdf]

Supplemental Figures

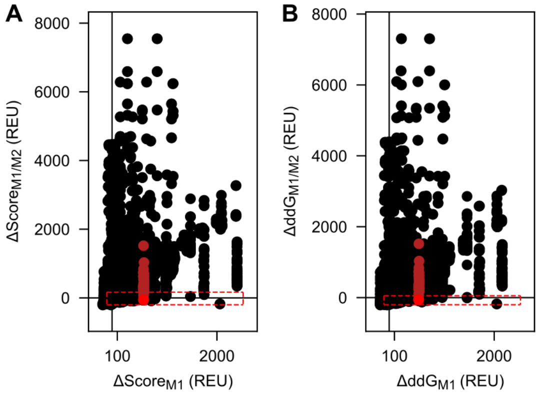

Fig. S1: Rosetta metrics for all single and pairwise 1WA3 trimer interface mutants. (**A**) ΔddG filter metric. (**B**) ΔScore metric. Dark red points correspond to the single mutation P114F. The bright red point corresponds to the double mutant P114F/F131V. The red dotted boxes represent cutoffs used to select mutants for testing.

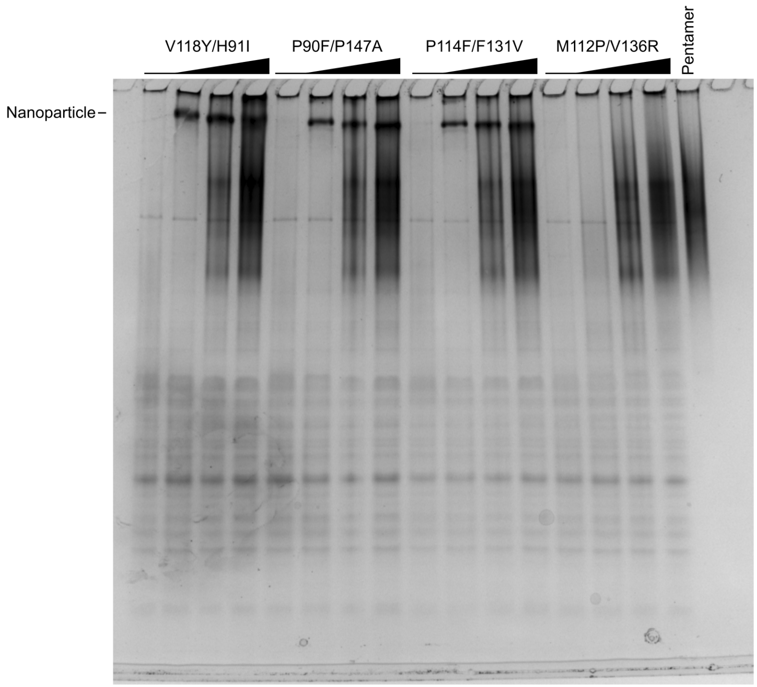

Fig. S2: Native-PAGE screen of double mutants. Recovery of trimer geometry was assayed by assembling double mutant I53-50A trimers in clarified *E. coli* lysates with purified I53-50B pentamer and evaluating the presence or absence of I53-50 nanoparticles by native PAGE. Black wedges indicate increasing pentamer concentration in each series of assembly reactions.

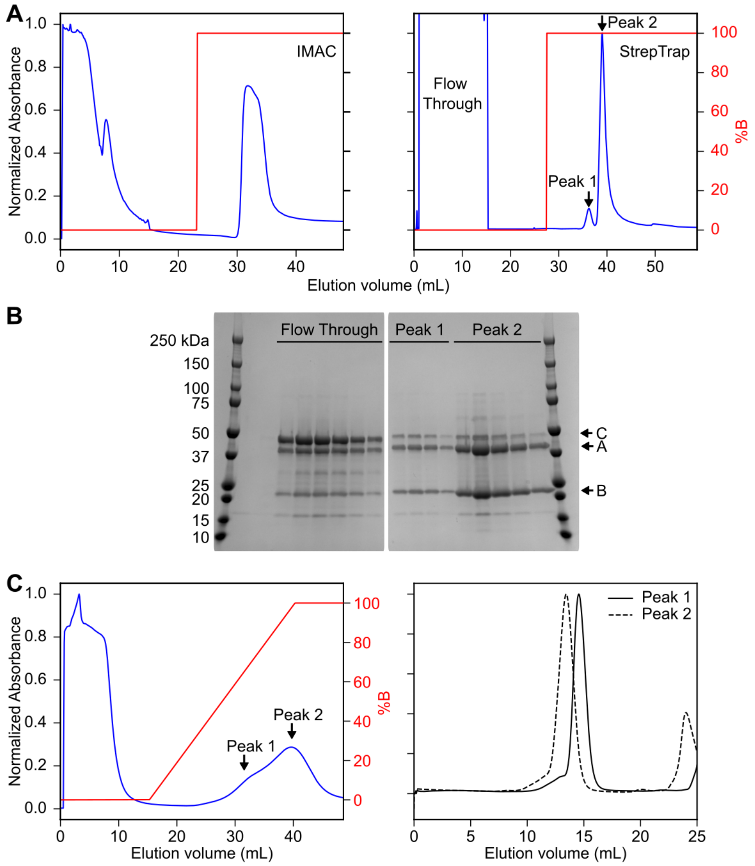

Fig. S3: Purification and characterization of "ABC" tricistronic and "AB" bicistronic constructs. (**A**) The ABC heterotrimer was purified by (*left*) IMAC with a step elution followed by (*right*) StrepTrap purification. The A chain contained a hexa-histidine and SUMO tag, the B chain contained a Strep tag, and the C chain contained sfGFP and avi tags. The eluate of this two-step purification method should therefore only contain trimers that include both the A and B chains. An optimal result would be equimolar amounts of the A, B, and C chains. (**B**) SDS-PAGE of the StrepTrap purification revealed that the eluate contained an excess of the A and B chains and less of the C chain. To test the ability of the A and B chains only to assemble into heterotrimers, we expressed an AB bicistronic gene and (**C**) purified the resulting proteins by (*left*) IMAC with a gradient elution. Two broad and overlapping peaks were observed. The leading half of the first peak and trailing half of the second peak were collected and (*right*) further purified by SEC. Peak 2 has a lower retention volume than peak 1, suggesting a difference in molecular weight. These results are consistent with assembly of an ABB heterotrimer (earlier IMAC elution, later SEC elution) and an AAB heterotrimer (later IMAC elution, earlier SEC elution). We confirmed this interpretation by native mass spectrometry (**Fig. 1G**)

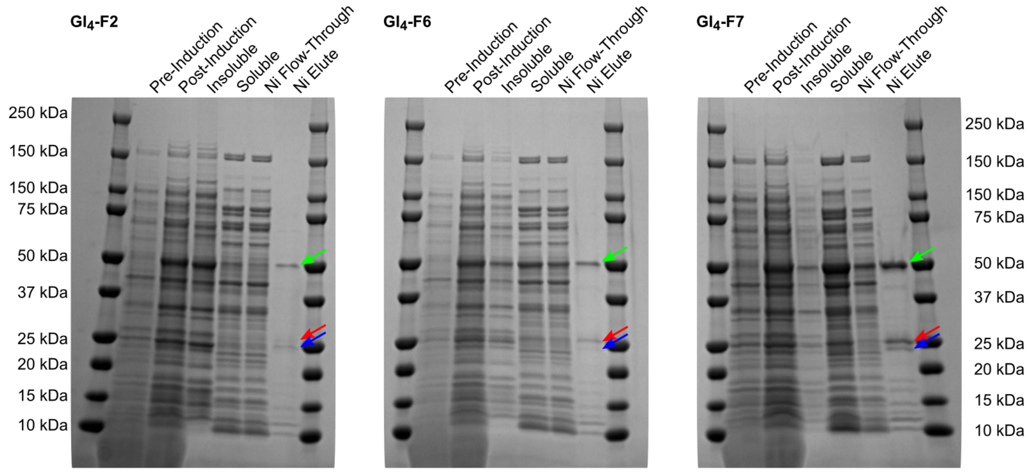

Fig. S4: SDS-PAGE of GI_4 designs by Ni^{2+} pull-down assay. Expression and screening by SDS-PAGE for GI_4 designs. Bands for chains A (green arrow), B (blue arrow), and C (red) arrow are indicated. The presence of all three bands in the Ni Elute lanes of GI_4-F6 and GI_4-F7 indicates interactions between the A, B, and C chains.

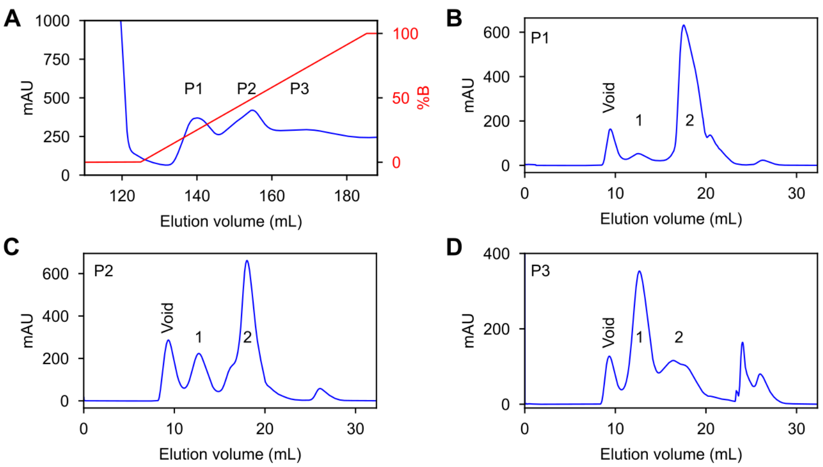

Fig. S5: Purification of heterotrimer components from A_{60}. (**A**) HisTrap elution chromatogram. Blue, absorbance at 280 nm; red, gradient elution. Peak 1 (P1) is predominantly ABB, P2 is predominantly AAB, and P3 is predominantly the A chain, which assembles into 60-subunit I3-01-like nanoparticles. (**B**) Superdex 200 Increase 10/300 chromatogram of P1 from the HisTrap elution. The first peak following the void volume (1) is predominantly I3-01-like nanoparticles and (2) is predominantly ABB heterotrimer. (**C**) Superdex 200 Increase 10/300 chromatogram of P2 from the HisTrap elution. (1) is predominantly I3-01-like nanoparticles and (2) is predominantly AAB heterotrimer. (**D**) Superdex 200 Increase 10/300 chromatogram of P3 from the HisTrap elution. (1) is predominantly I3-01-like nanoparticles and (2) is predominantly AAB heterotrimer.

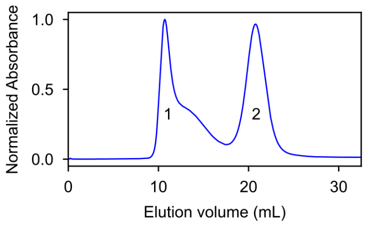

Fig. S6: Purification of GI$_4$-F7 by SEC. SEC purification of GI$_4$-F7 on a Sephacryl S-500 HR 10/300 GL column. Peak 1 contains the assembly while peak 2 is residual homotrimer component.

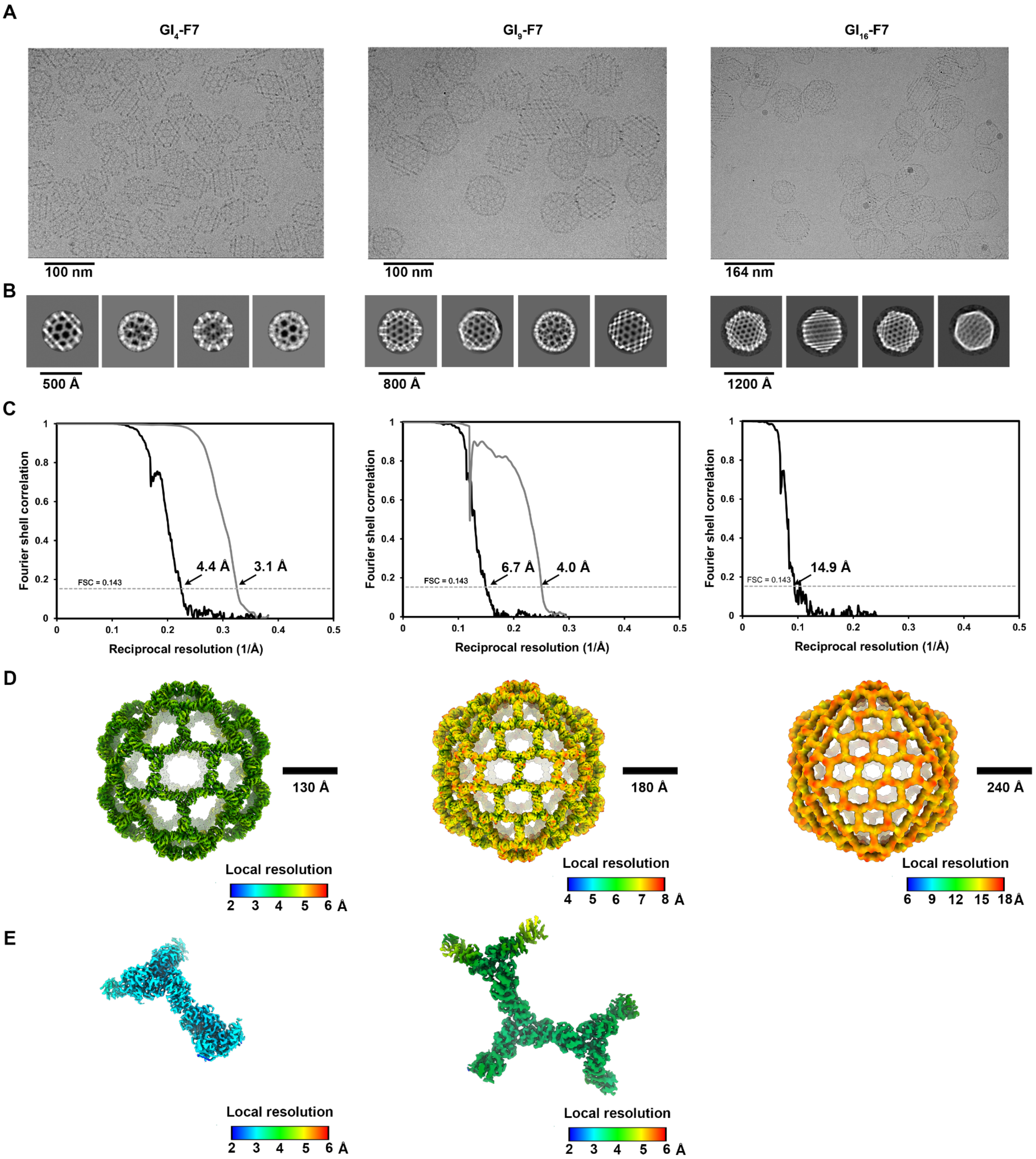

Fig. S7: CryoEM data processing. (**A-B**) Representative electron micrographs (A) and 2D class averages (B) of GI_4-F7 (left), GI_9-F7, (middle) and GI_{16}-F7 (right). (**C**) Gold-standard Fourier shell correlation curves for the 3D reconstructions of GI_4-F7 (left), GI_9-F7 (middle) and GI_{16}-F7 (right) (black line) and locally refined asus (gray lines). (**D-E**) Local resolution maps calculated using cryoSPARC for (D) the 3D reconstructions of GI_4-F7 (left), GI_9-F7 (middle), and GI_{16}-F7 (right) as well as (E) the locally refined ASUs of GI_4-F7 (left) and GI_9-F7 (middle). The 0.143 cutoff is indicated by a horizontal dashed line.

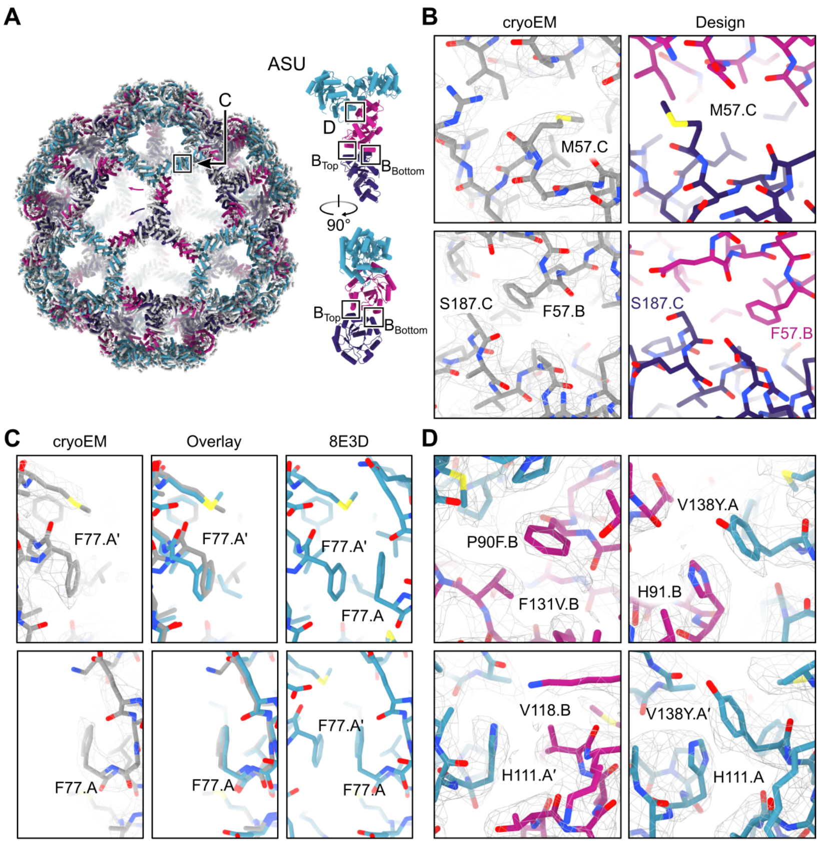

Fig. S8: Structural details of GI$_4$-F7. (**A**) Alignment of the complete cryoEM model to the design model. Major rigid-body DoF deviations are indicated with arrows. Two views of the ASU are shown. Approximate locations of each inset (B, C, and D) are indicated. (**B**) Comparison between the cryoEM model (left) and design model (right) of the newly designed nanoparticle (B-C) interface. *Top row*, M57 on the CCC-homotrimer changes rotamer to occupy a void in the interface in the design model. *Bottom row*, F57 on the B chain of the AAB heterotrimer packs against S187 of the CCC homotrimer in the cryoEM model, instead of A190 in the CCC homotrimer as in the design model. (**C**) Comparison of the I3-01 (A-A) interface observed in the cryoEM model to a previously published structure (PDB ID 8ED3). *Top row*, slight rigid-body deviations from perfect two-fold symmetry in one copy of the A chain. *Bottom row,* very little deviation from perfect two-fold symmetry (**D**) Details of the density maps in the regions of the pseudosymmetry-generating mutations within the AAB heterotrimer interface.

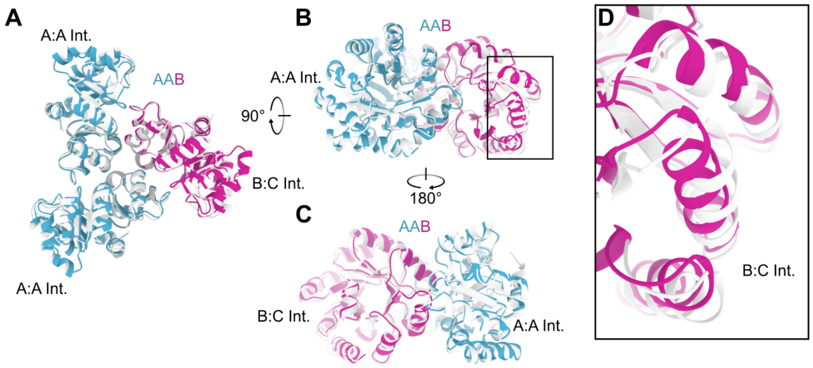

Fig. S9: Comparison of the AAB heterotrimer design model to the cryoEM model from GI_4-F7. Alignment of the AAB heterotrimer cryoEM model to the design model is viewed (**A**) from the top, towards the center of the nanoparticle along the three-fold symmetry axis; (**B**) from the side, tangential to the nanoparticle surface; and (**C**) from the other side, tangential to the nanoparticle surface. The position of the A:A and newly designed B:C interfaces are indicated. (**D**) Detail of the B side of the B:C interface, highlighting the most significant deviations from the design model.

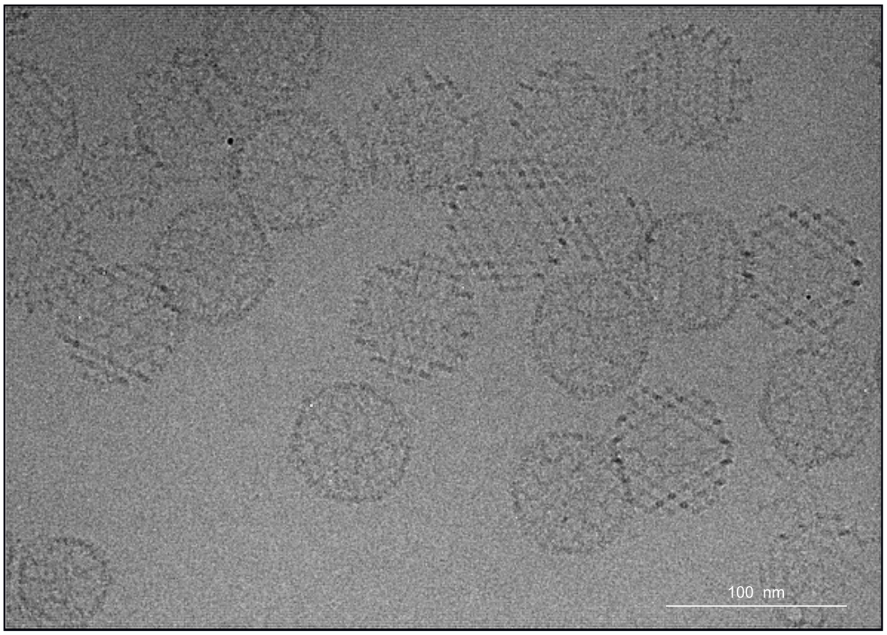

Fig. S10: Field view micrograph of GI_9-F7. CryoEM field view micrograph of samples enriched for GI_9-F7 by SEC purification. Both GI_9-F7 (large particles) and GI_4-F7 (e.g., bottom-left corner) are clearly visible.

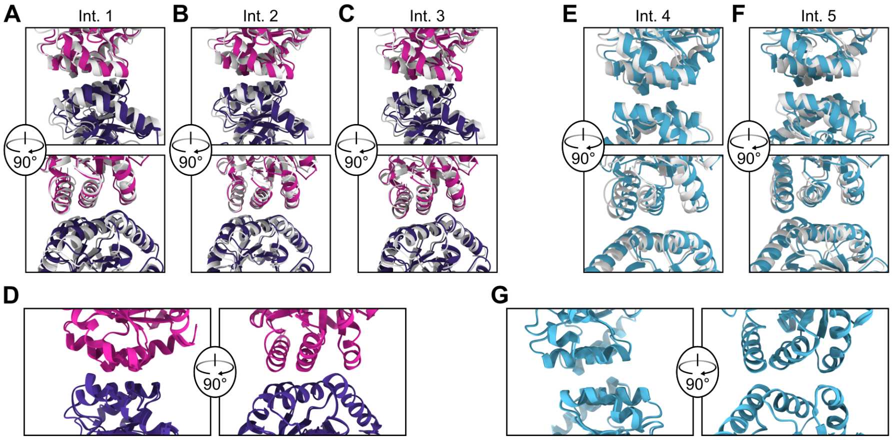

Fig. S11: Structural details of GI_9-F7. (**A-C**) Alignment of the GI_9-F7 design model chain B (magenta) and chain C (purple) protein-protein interface to the corresponding chains of the cryoEM model (gray). Each of the three interfaces between B and C chains in the ASU are shown. (**D**) The protein-protein interface between chain B and C from the cryoEM model of GI_4-F7 (light colors) aligned to the same interface from the cryoEM model of GI_9-F7 (dark colors). (**E**) Alignment of design model to the cryoEM model for the I3-01 interface in the pentasymmetron and (**F**) disymmetron. (**G**) Alignment of the I3-01 interface from the cryoEM models of GI_4-F7 (light blue) and GI_9-F7 (dark blue).

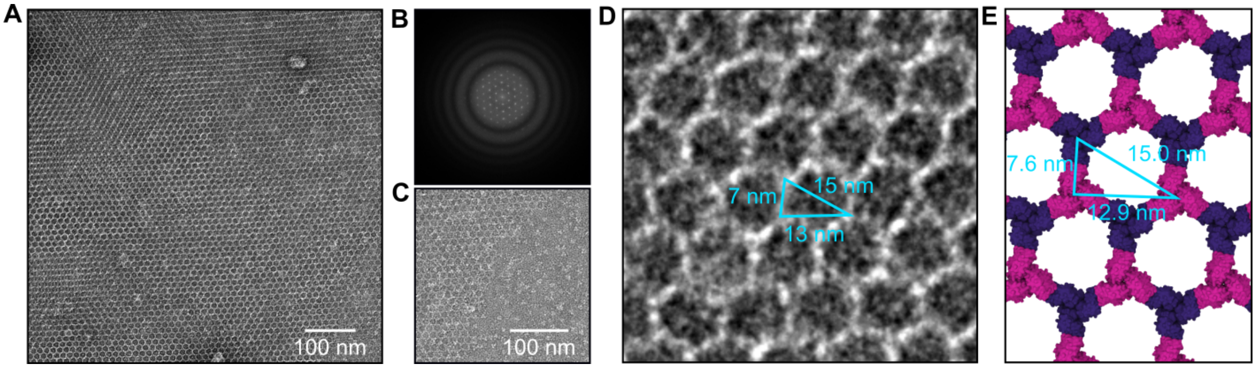

Fig. S12: Hexagonal 2D array characterization by negative stain EM. (**A**) An example of the regular hexagonal array formed by mixing BBB and CCC homotrimers by negative stain EM. (**B**) Power spectrum of the micrograph shown in panel A, confirming the periodic arrangement of the array. (**C**) The edge of the array is jagged, with free trimeric components visible. (**D**) Measurement of the array dimensions are consistent with (**E**) the design model.

Supplemental Tables

Supplementary Table S1: Table of all single and double mutants and outcome.

Single mutant	Assembles in lysate?	Single mutant	Assembles in lysate?	Single mutant	Assembles in lysate?
R17Y	Yes	T113V	Yes	**F140K**	**No**
T69E	Yes	P114Q	Yes	F140H	Yes
T69G	Yes	P114S	Yes	F140W	Yes
T71A	Yes	**P114F**	**No**	A143D	Yes
T71D	Yes	T115K	Yes	A143E	Yes
T71E	Yes	T116C	Yes	A143Y	Yes
T71K	Yes	T116D	Yes	P147A	Yes
T71L	Yes	T116G	Yes	P147D	Yes
T71N	Yes	T116L	Yes	P147H	Yes
T71Y	Yes	T116M	Yes	P147M	Yes
P90E	Yes	T116N	Yes	P147R	Yes**
**P90F**	**No**	T116Q	Yes	P147S	Yes
P90K	Yes	T116S	Yes	F148C	Yes
P90Q	Yes	T116T	Yes	F148E	Yes
P90R	Yes	**T116W***	**No**	F148H	Yes
H91C	Yes	V118H	Yes	F148Q	Yes
H91D	Yes	V118K	Yes	F148Y	Yes
H91F	Yes	V118M	Yes		
H91G	Yes	V118N	Yes		
H91I	Yes	V118S	Yes		
H91S	Yes	V118W	Yes		
D93R	Yes	**V118Y**	**No**		
**M112P**	**No**	K122D	Yes		
M112Q	Yes	K122E	Yes		
M112V	Yes	F131E	Yes**		
T113D	Yes	E134G	Yes		
T113F	Yes	E134K	Yes		
T113I	Yes	V135P	Yes		
T113L	Yes	V136R	Yes		
T113M	Yes	V136Y	Yes		
T113P	Yes	V136W	Yes		
T113Q	Yes	Q139E	Yes		

Double mutant	Assembles in lysate?
M112P/V136R	No
P90F/P147A	Yes
P114F/F131V	Yes
V118Y/H91I	Yes

* Mutant at three-fold position required screening in a tricistronic gene construct and was not pursued further.

** Did not assemble in one replicate or had a weak assembly band.

Supplementary Table S2. Amino acid sequences for novel proteins used in this study.

>Pseudosymmetric I53-50A A Chain
MGHHHHHHHHHHGSLQDSEVNQEAKPEVKPEVKPETHINLKVSDGSSEIFFKIKKTTPLRRLMEAFAKRQGKEMDSLRF
LYDGIRIQADQAPEDLDMEDNDIIEAHREQIGGSEKAAKAEEAARKMEELFKKHKIVAVLRANSVEEAIEKAVAVFAGGVHL
IEITFTVPDADTVIKALSVLKEKGAIIGAGTVTSVEQCRKAVESGAEFIVSPHLDEEISQFCKEKGVFYMPGVMTPTELYKA
MKLGHDILKLFPGEVVGPQFVKAMKGPFPNVKFVPTGGVNLDNVCKWFKAGVLAVGVGKALVKGKPDEVREKAKKFVK
KIRGCTE

>Pseudosymmetric I53-50A B Chain
MKMEELFKEHKIVAVLRANSVEEAISKALAVFAGGVHLIEITFTVPDADQVIKELEFLKEAGAIIGAGTVTSVEQCREAVESG
AEFIVSFHLDEEISQFCKEEGVFYMPGVMTPTELVKAMKLGHTILKLVPGEVVGPQFVEAMKGPFPNVKFVPTGGVNLD
NVCEWFEAGVLAVGVGSALVEGEPAEVAELAIRFVEKIRGCTEGSGWSHPQFEK

>Pseudosymmetric I53-50A C Chain
MKGEELFTGVVPILVELDGDVNGHKFSVRGEGEGDATNGKLTLKFICTTGKLPVPWPTLVTTLTYGVQCFARYPDHMKQ
HDFFKSAMPEGYVQERTISFKDDGTYKTRAEVKFEGDTLVNRIELKGIDFKEDGNILGHKLEYNFNSHNVYITADKQKNGI
KANFKIRHNVEDGSVQLADHYQQNTPIGDGPVLLPDNHYLSTQSVLSKDPNEKRDHMVLLEFVTAAGITHGMDELYKGG
SGGSGGKMEELFKKHKIVAVLRANSVEEAIEKAVAVFAGGVHLIEITFTVPDADTVIKALSVLKEKGAIIGAGTVTSVEQCRK
AVESGAEFIVSPILDEEISQFCKEKGVFYMPGVMTFTELVKAMKLGHTILKLFPGEVVGPQFVKAMKGAFPNVKFVPTGG
VNLDNVCEWFKAGVLAVGVGSALVKGTPDEVREKAKAFVEKIRGCTEGSGLNDIFEAQKIEWHE

>Pseudosymmetric GI$_T$ A Chain
MGSHHHHHHGSEKAAKAEEAARKMEELFKEHKIVAVLRANSVEEAKKKALAVFLGGVHLIEITFTVPDADTVIKELSFLKE
MGAIIGAGTVTSVEQCREAVESGAEFIVSPHLDEEISQFCKEEGVFYMPGVMTPTELYKAMKLGHTILKLFPGEVVGPQF
VEAMKGPFPNVKFVPTGGVNLDNVCEWFEAGVLAVGVGSALVEGTPVEVAEKAKAFVEKIEGCTE

>Pseudosymmetric GI$_T$ B Chain
MKMEELFKEHKIVAVLRANSVEEAISKALAVFAGGVHLIEITFTVPDADQVIKELEFLKEAGAIIGAGTVTSVEQCREAVESG
AEFIVSFHLDEEISQFCKEEGVFYMPGVMTPTELVKAMKLGHTILKLVPGEVVGPQFVEAMKGPFPNVKFVPTGGVNLD
NVCEWFEAGVLAVGVGSALVEGEPAEVAELAIRFVEKIRGCTE

>Homotrimeric GI$_T$ BBB
MKMEELFKEHKIVAVLRANSVEEAISKALAVFAGGVHLIEITFTVPDADQVIKELEFLKEAGAIIGAGTVTSVEQCREAVESG
AEFIVSPHLDEEISQFCKEEGVFYMPGVMTPTELVKAMKLGHTILKLFPGEVVGPQFVEAMKGPFPNVKFVPTGGVNLD
NVCEWFEAGVLAVGVGSALVEGEPAEVAELAIRFVEKIRGCTELEHHHHHH

>Homotrimeric GI$_T$ CCC
MKMEELFKEHKIVAVLRANSREEAIEIALAVFAGGVHLIEITFTVPDADEVIKRLEMLKRAGAIIGAGTVTSVEQCREAVESG
AEFIVSPHLDEEISQFCKEEGVFYMPGVMTPTELVKAMKLGHTILKLFPGEVVGPQFVEAMKGPFPNVKFVPTGGVNLD
NVCEWFEAGVLAVGVGSALVEGKPSEVAEKARRFVKKIRGCTEGSLEHHHHHH

Appended sequences including SUMO, GFP, and deca- or hexa-histidine, avi- and strep- tags are underlined.
Pseudosymmetrizing mutations are highlighted.

Supplementary Table S3. CryoEM data collection and refinement statistics.

	GI_4-F7<br><br>EMD XXXX	GI_4-F7 (local refinement)<br>PDB XXXX<br>EMD XXXX	GI_9-F7<br><br>EMD XXXX	GI_9-F7 (local refinement)<br>PDB XXXX<br>EMD XXXX	GI_{16}-F7<br><br>EMD XXXX
**Data collection and processing**					
Magnification	105,000	105,000	105,000	105,000	64,000
Voltage (kV)	300	300	300	300	300
Electron exposure ($e^{-}/Å^2$)	60	60	60	60	37
Defocus range (µm)	-0.5 - -2.5	-0.5 - -2.5	-0.5 - -2.5	-0.5 - -2.5	-0.5 - -3.5
Pixel size (Å)	0.843	0.843	0.843	0.843	1.42
Symmetry imposed	I	C1	I	C1	I
Initial particle images (no.)	154,574		18,611		1,226
Final particle images (no.)	120,979	984,020	1,956	795,360	1,083
Map resolution (Å)	4.4	3.1	6.7	4.0	14.9
FSC threshold	0.143	0.143	0.143	0.143	0.143
**Refinement**					
Map resolution (Å)<br>FSC threshold		0.143		0.143	
Map sharpening *B*factor ($Å^2$)	-269	-143	-530	-138	
Model composition<br>Non-hydrogen atoms<br>Protein residues<br>Ligands		5687<br>813<br>0		9161<br>1827<br>0	
B factors (Å2)<br>Protein<br>Ligand		15.53		73.75	
**Validation**					
MolProbity score		1.9		1.06	
Clashscore		5.86		0.71	
Poor rotamers (%)		2.94		0.13	
Ramachandran plot					
Favored (%)		96.52		98.95	
Allowed (%)		3.11		0.5	
Disallowed (%)		0.37		0.55	

Supplementary Table S4: Deviations observed in the cryoEM reconstruction of GI_4-F7 compared to the design model.

Component	Axis	Translation (Å)	Rotation (Degrees)
Pentasymmetron	5-fold	5.8	5.9
AAB-Heterotrimer	// Local 3-fold	1.1	1.3
	⊥ Local 3-fold	1.6	–
	AAB:AAB Interface	–	1.6, -2.8
	AAB:CCC Interface	–	-0.2
Trimer	3-fold	4.0	12.4

Supplementary Table S5: Deviations observed in the cryoEM reconstruction of GI_9-F7 compared to the design model.

Component	Axis	Translation (Å)	Rotation (Degrees)
Pentasymmetron AAB heterotrimer	Icosahedral 5-fold	7.7	7.2
	// * Local 3-fold	1.3	1.8
	⊥ Local 3-fold	1.9	--
	AAB:AAB Interface	--	1.9, -3.6
	AAB:CCC Interface	--	0.3
Disymmetron ABB heterotrimer	Icosahedral two-fold	10.0	8.2
	// Local 3-fold	0.4	2.3
	⊥ Local 3-fold	1.3	--
	ABB:ABB Interface	--	-1.6
	ABB:CCC Interface	--	-0.8, 3.7
3× Trimer CCC homotrimer	Icosahedral 3-fold	6.5	5.4
	// Local 3-fold	0.0	-6.5
	⊥ Local 3-fold	1.23	--
	CCC:ABB Interface	--	0.3, -1.8
	CCC:AAB Interface	--	-0.6*

*The symbol // indicates deviations parallel to the indicated symmetry axis, ⊥ indicates deviations perpendicular to the indicated axis.

Supplementary Table S6: DLS results obtained from assembly reactions corresponding to T numbers 4 to 100.

T	Mean Z-Ave. dia. (nm)	StdDev Z-Ave. dia. (nm)	PDI
4	47.5	0.4	0.048
9	69.9	0.5	0.154
16	95	1	0.121
25	113.2	0.5	0.127
36	129	2	0.134
49	148	2	0.136
64	162	1	0.136
81	174	1	0.126
100	189	1	0.133
